# Inspiration and encounters: Carl Woese and my 30‐year research journey

**DOI:** 10.1002/mlf2.12050

**Published:** 2022-12-18

**Authors:** Yoshizumi Ishino

**Affiliations:** ^1^ Department of Bioscience and Biotechnology, Graduate School of Bioresource and Bioenvironmental Sciences Kyushu University Fukuoka Japan

I clearly remember the day in February 1998 when I met Professor Carl Woese for the first time at his laboratory in the Department of Microbiology, the University of Illinois at Urbana–Champaign. Prof. Woese kindly gave me the reprint of his seminal review paper with his signature (*Microbiological Reviews* in 1987)^1^. A veritable treasure, I keep this paper in my office, having started research on the molecular biology of Archaea in 1992 and having always aspired to meet him someday. In this essay, I highlight the remarkable achievements of Carl Woese and share my memories of him, while looking back at my own research on Archaea and related aspects in Japan.

## DAWN OF MOLECULAR BIOLOGY STUDIES IN ARCHAEA

When I was a postdoctoral fellow at Yale University in 1987, polymerase chain reaction (PCR) technology began to be applied practically in 1988^2^. I came back to Japan in 1989 and set up my research group at a biotech company, Takara Shuzo (currently, Takara Bio). Our task was to develop useful reagents and instruments for genetic engineering, and PCR was the main focus of our work at that time. We made a highly purified Taq DNA polymerase and developed it for use as the standard enzyme for PCR^3^. Because of my job profile, I became interested in thermophilic organisms to produce thermostable enzymes. For the first time, I got to see Achaea because most of the hyperthermophilic organisms identified by that time belonged to that domain.

In graduate school, I started my research on restriction endonuclease and DNA ligase, and I wrote theses for my master and doctoral degrees on these works. Since then, my research has consistently been about enzymes and protein factors acting on DNA and RNA. Among DNA polymerases, bacterial PolI differed greatly from eukaryotic Polα in properties, and they were classified into two families, PolI‐like and Polα‐like. When I determined the nucleotide sequence of the *polB* gene of *Escherichia coli*, it was surprising that the deduced amino acid sequence was similar to that of eukaryotic Polα^4^. It was fascinating that prokaryotes have a eukaryotic DNA polymerase, but *polB* is not essential for viability, likely being involved in the repair of certain types of DNA damage. During that study, I focused on the report by Patrick Forterre et al.^5^, according to which aphidicolin, a specific inhibitor of Polα‐like DNA polymerases, inhibits the growth and nucleotide incorporation of a halophilic archaeon, *Halobacterium halobium*. This meant that a Polα‐like DNA polymerase works for DNA replication in this archaeon. My deep interest in DNA polymerase in hyperthermophilic archaea led me to attempt to purify a DNA polymerase from the cell extract of *Pyrococcus furiosus* in 1990. Then, we determined the sequence of 11 amino acids from the N‐terminus of the purified protein. Using this information, I designed a mixed primer for PCR. Because of the lack of further information on the amino acid sequence of the protein, a genomic DNA library was made by restriction enzyme cleavage, followed by ligation of a cassette oligo DNA. We used the sequence of the cassette DNA for designing the reverse primer. Then, we succeeded in amplifying a specific DNA fragment of the *pol* gene. The region upstream of the initiation codon ATG was also amplified by the same PCR strategy. Finally, we determined the entire nucleotide sequence of the gene and the transcription start site. The deduced amino acid sequence clearly showed a Polα‐like sequence^6^. DNA polymerases from hyperthermophilic archaea became the targets of archaeal molecular biology at that time, and two other groups published papers on *pol* genes, that is, with deduced amino acid sequences from *Thermococcus litoralis* by Francine Perler et al.^7^ in the United States and from *Sulfolobus solfataricus* by Francesca Pisani et al.^8^ in Italy, just before our findings were published^6^. These results were the first reports demonstrating the entire sequences of the archaeal DNA polymerases and clearly showed that archaea have eukaryotic α‐like DNA polymerases. At the same time, we also studied another hyperthermophilic archaeon, *Pyrodictium occultum*, and tried to clone a gene encoding a family‐B DNA polymerase using a set of mixed primers based on the conserved motifs A and C in this family. We succeeded, but surprisingly obtained two different genes, both of which had Polα‐like sequences and belonged to family B. This was the first demonstration of two family‐B DNA polymerases in an archaeal cell^9^. Because all three eukaryotic replicases, Polα, δ, and ε, belong to family B, it was an exciting prospect that archaeal DNA replication machinery is closely similar to that of Eukarya but not to that of Bacteria.

## DIGGING DEEPER INTO ARCHAEAL MOLECULAR BIOLOGY

**Figure 1:**
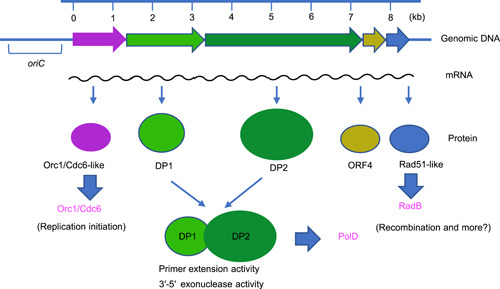
Identification of the operon including genes for PolD, Orc1/Cdc6‐like, and Rad51‐like proteins from *Pyrococcus furiosus*. Both DP1 and DP2 are required for both 5′‐3′ synthesizing and 3′‐5′ exonuclease activities. The replication origin oriC was identified in the upstream region of this operon later.

I was keen to determine how many DNA polymerases archaeal cells have, and I, therefore, continued to purify other enzymes. We found three fractions containing nucleotide incorporation activity after anion‐exchange chromatography of *P. furiosus* cell extracts. We found an aphidicolin‐resistant fraction among them and performed further purification^10^. However, it was difficult to purify sufficient amounts for analyses. My team's unique strategy to clone the genes encoding DNA polymerases was to construct a gene library from *P. furiosus* genomic DNA and prepare the cell extracts from different *E. coli* transformants. These cell extracts were treated at 94°C for 20 min for denaturing *E. coli* proteins. The supernatants of this heat treatment provided heat‐stable proteins produced by *P. furiosus* genes. In this way, we successfully isolated the genes for the second DNA polymerase (PolII), which consisted of two proteins, DP1 and DP2^11^. It was believed that DNA polymerases are conserved among living organisms, being of either bacterial type or eukaryotic type. However, it was striking that the deduced amino acid sequences of PolII completely differed from those of any known DNA polymerase^11^. In addition, we discovered a protein whose sequence was similar to that of eukaryotic Orc1 and Cdc6. These proteins are related to the DNA replication initiator of Eukarya. Furthermore, we discovered a protein similar to the eukaryotic Rad51 recombinase. These eukaryotic‐like proteins were encoded by genes upstream and downstream of the gene encoding PolII, and they were transcribed together in an operon^11^ (Figure 1). This discovery marked the peak of my interest in archaea, and I strongly felt like devoting my research career to basic molecular biology, especially DNA replication and recombinational repair in Archaea. We wanted to publish these data in a popular international journal, but we had a hard time convincing editors of the merit of the work. Five journals rejected our manuscript because the editors did not believe our discovery of a new DNA polymerase without sequence homology with any known DNA polymerase.

During those challenging times, I attended the international meeting, *Thermophiles 1996*, at the University of Georgia, United States, and presented our data as a poster. There, I met Prof. Forterre for the first time. It was an unforgettable experience. He was the first to visit my poster and listened to my explanation intently. Since then, he has been a close friend, constantly supporting and encouraging my research. That year, the first total genome sequence of an archaeal cell was published^12^, and there was much buzz around the genome sequence of the methanogenic archaeon, *Methanococcus jannaschii*. This organism has only one DNA polymerase, which is a Polα‐like (family B) DNA polymerase, in its genome. No open reading frame encoding any other DNA polymerase‐like sequence was found. At that time, it was known that *E. coli* has three DNA polymerases (PolI, II, III), and humans have five (Polα, β, γ, δ, ε). Therefore, it was rather surprising that the archaeal cell gets by with only a single DNA polymerase^12^, ^13^. We were still struggling to publish findings on a new DNA polymerase, although we were probably the only ones at that time who knew the sequence of the new DNA polymerase. We looked for genes encoding a homolog of our enzyme and found both genes for DP1 and DP2 in the *M. jannaschii* genome. I wanted to prove that these genes actually encode an active DNA polymerase, and therefore, I began looking for a laboratory in Japan where *M. jannaschii* could be cultivated. That is when I thought of Prof. Yosuke Koga in the University of Occupational and Environmental Health, Kitakyushu, a pioneering scientist of archaeal research in Japan. I had already read a textbook on Archaea written by Prof. Koga in Japanese in 1988. It was the only book explaining Archaea at that time. I wrote a letter to him asking about an opportunity to cultivate *M. jannaschii*. He kindly accepted my request to visit his laboratory and even provided *M. jannaschii* cells. I proceeded to prepare its genomic DNA and clone the genes for DP1 and DP2. It was also interesting that the two genes were located separately in the *M. jannaschii* genome, unlike the operon structure in the *P. furiosus* genome. Finally, we demonstrated the nucleotide incorporation activity and the 3 → 5 exonuclease activity from the proteins produced in *E. coli* cells. Both activities were detected from only the mixture of the two proteins but not either DP1 or DP2 alone. This result was soon submitted, and finally published in the *Journal of Bacteriology* ^14^, after rejections from two other journals.

## FAMILY D DNA POLYMERASE

Isaac Cann, who is now a professor at the University of Illinois at Urbana–Champaign, joined my group at Biomolecular Engineering Research Institute (BERI) in Osaka in 1997. Isaac and I attended the Keystone meeting of Archaea held in Taos, New Mexico, in 1998. Next, we visited the University of Illinois and asked Prof. Woese if we could visit his laboratory. He kindly accepted, and I was able to finally meet him. We introduced our novel DNA polymerase found in *P. furiosus* and *M. jannaschii* and discussed this with him. He was very receptive and encouraged us to continue our archaeal research in this direction. Moreover, he gave me an autographed reprint of his influential review paper^1^. We came back to Japan impressed and inspired.

After the genome of *M. jannaschii* was sequenced, the total genome sequences of *Archaeoglobus fulgidus*, *Methanobacterium thermoautotrophicum*, and *Pyrococcus horikoshii* were continuously reported. We found the genes for DP1 and DP2 in these archaeal genomes as well and proposed a new family of DNA polymerases that may be a replicase, at least, in *Euryarchaeota*, a subdomain of Archaea. Prof. Woese communicated our paper for publication in the *Proceedings of the National Academy of Science USA* in 1998^15^. In our subsequent review paper, we proposed “family D” for these new archaeal DNA polymerases^16^. Since our discovery of these archaea‐specific DNA polymerases, other research groups started to work on this enzyme, namely, from *P. horikoshii* by Ikuo Matsui et al. in Japan and from *P. abyssi* by Jean‐Paul Raffin, Joël Querellou, et al. in France. One question remained, whether PolD is distributed across the whole archaeal domain because no total sequence had been reported for any crenarchaeal organism. Instead, we identified two family‐B DNA polymerases in the crenarchaeon *Aeropyrum pernix* ^17^, followed by *P. occultum* ^9^, as described above. The answer to this question was obtained when the total genome sequences of *A. pernix* and *S. solfataricus* were reported. It had been proposed that Polε and Polδ perform nascent DNA synthesis of the leading strand and lagging strand, respectively, and therefore, I expected that two family‐B DNA polymerases in *Crenarchaeota* work on the leading strand and lagging strand, just like in the eukaryotic system. On the other hand, PolD instead of PolB functions to synthesize one of the nascent strands to proceed with replication.

## DNA REPLICATION MECHANISM IN ARCHAEA

I was captivated by DNA replication in Archaea and went on to purify replication‐related proteins, including Orc1/Cdc6, Mcm, primase (p41‐p46 or PriL/PriS), DNA ligase, Fen1, PCNA, RFC (1RfcL‐4RfcS), GINS (2Gisn51‐2Gins23), and GAN from *P. furiosus* to construct an in vitro DNA replication system. In 2000, Patrick Forterre predicted that the *Pyrococcus* genome replicated from a single origin and experimentally proved this prediction in the *P. abyssi* genome^18^. I joined his laboratory at the University of Paris‐Sud in 2000, and we identified the*oriC* region in *P. abyssi* genome together^19^. Interestingly, *oriC* was located just upstream of the gene for Orc1/Cdc6, a putative replication initiator in Archaea. The relative positional relationships between *oriC* and the gene for the initiator were similar to that of bacterial *oriC* and *dnaA*. These results showed that Archaea is really distinct from Bacteria and Eukarya, possessing a bacterial circular genome with one *oriC* and replication machinery similar to that of Eukarya.

Shortly after that, in 2002, along with Patrick Forterre, Jocelyne DiRuggiero (University of Maryland), and Yoshie Harada (Tokyo Metropolitan Medical Institute), I was elected as a Human Frontier Science Program (HFSP) Grant awardee for the first archaeal HFSP research proposal “Structural and functional analyses of the initiation mechanism of archaeal DNA replication.” This collaboration increased my interest in Archaea manifold.

## 30TH ANNIVERSARY OF THE DISCOVERY OF THE THIRD DOMAIN OF LIFE

**Figure 2:**
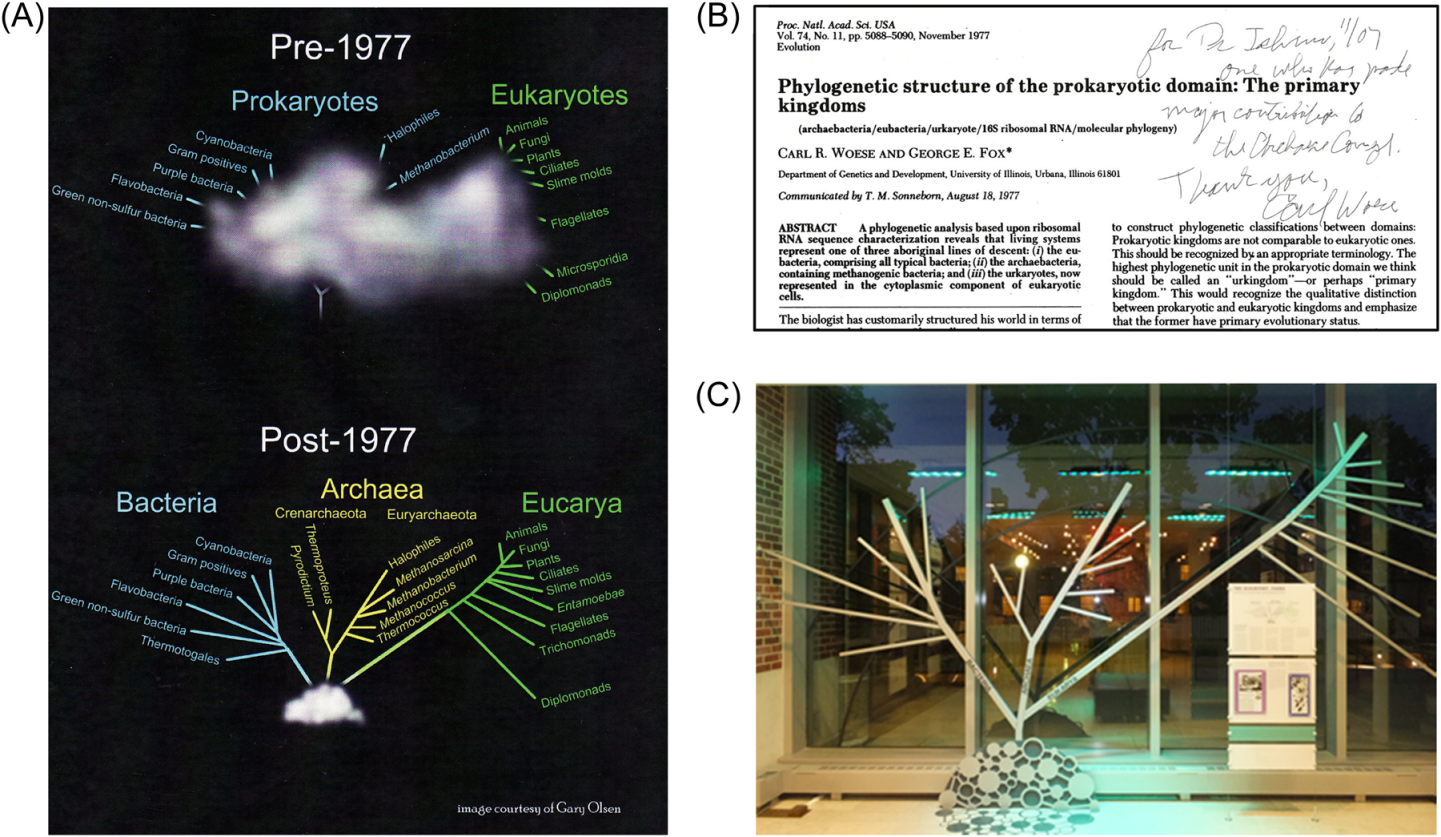
Memories with Carl Woese. (A) Symbol figure for the 30th Anniversary on the Discovery of Archaea “Hidden before our eyes” (November 2007, University of Illinois). (B) Reprint of the memorable paper showing archaea for the first time with an encouraging message by Carl Woese. (C) The Carl Woese Institute for Genomic Biology displays a model of a phylogenetic tree showing the three domains of life (at the University of Illinois at Urbana–Champaign).

In 2007, a memorial symposium for the 30th anniversary of the discovery of the third domain of life, titled “Hidden Before Our Eyes,” was held at the University of Illinois campus (Figure 2A). Prof. Koga and I were the only two Japanese participants. It was wonderful to spend two full days with a number of scientists involved in archaeal research. Prof. Woese looked so happy to be amidst this commemoration of his momentous discovery. He invited us to his laboratory and showed us the real raw data indicating that the organism he had analyzed was indeed distinctly different from Bacteria and Eukarya. He analyzed the structure of 16S rRNA by the footprinting method using nuclease T1 digestion. His data clearly showed that the cleavage pattern of his methanogen sample was completely different from those of known Bacteria. Prof. Woese then gave me another document that really moved me—a reprint of his memorial paper showing his first proposal of Archaea, published in 1977 in the *Proceedings of the National Academy of Sciences USA* ^20^, with his signature and a very warm message (Figure 2B).

For the 30th anniversary of the discovery of Archaea, I had planned to publish a specific issue of Tanpakushitu, Kakusan, and Koso (Proteins, Nucleic Acids, and Enzymes in Japanese), which is a popular monthly journal for the basic life sciences published in Japanese, by negotiating with a publisher. I asked several Japanese scientists, including Akihiko Yamagishi, Takuro Nunoura, Ken Takai, Harry Atomi, Tadayuki Imanaka, Yosuke Koga, Toshihisa Oshima, and their colleagues, to write review articles on their research subjects in Archaea. I also wrote a review of DNA replication and repair in Archaea with Sonoko Ishino. Tairo Oshima and Masahiro Kamekura wrote short column articles for this specific issue, and the issue was finally published in February 2009. I am convinced that this publication helped boost Archaea research in Japan.

## PROMOTION OF RESEARCH ON ARCHAEA

Next, I also wanted to publish a textbook for the study of Archaea in Japanese. Only two books on Archaea written in Japanese were available before I planned one in 2017. One was *Archaebacteria*, which was written by Yosuke Koga in 1988, and the other was *Archaea Biology*, edited by Yosuke Koga and Masahiro Kamekura in 1998. Twenty years on since *Archaea Biology*, we needed a new book. We published a new textbook in 2017, *Biology of Archaea*, which was edited by Harry Atomi (Kyoto University) and myself, and it included our most recent knowledge till that point. We even asked many young Japanese scientists to contribute text from their own archaeal research. Currently, it is the only Japanese textbook covering all the research progress on Archaea to date.

Our senior people established a small society in 1988, the “Japan Society for Archaea”, and we hold annual meetings in different locations across the country. At the 34th annual meeting in Fukuoka in July, 2022, about 60 eminent researchers visited Fukuoka and spent two meaningful days together after 3 years. It was my third time organizing the annual meeting after the 14th meeting in Osaka in 2001 and 19th meeting in Fukuoka in 2006. Any face‐to‐face meeting had been prohibited for 2 years because of COVID‐19, and therefore, it was heartening to interact closely with many Japanese scientists in the archaeal community.

## RECENT TOPICS OF RESEARCH ON REPLISOME OF ARCHAEA

We now know that most archaea, except crenarchaea, have two DNA polymerases, PolB and PolD. However, PolD is probably a replicase because thus far, neither the *dp1* or *dp2* gene has been successfully deleted from the genome, although *polB* can be deleted without any growth defect for several archaea^21^, ^22^. The evolutionary origin and the three‐dimensional (3D) structure of PolD have been a mystery for many years because it is not easy to purify it and DP2 has no sequence homology with other DNA polymerases. Our study on *Thermococcus kodakkarensis* and study on *Pyrococcus abyssi* by the Sauguet group (Inst. Pasteur) showed remarkable progress in the archaeal replisome in recent years. Although progress in crystal structure analysis has been slow, the development of cryo‐electron microscopy technology (cryoEM) led to the successful analysis of the 3D structure of the whole molecule of PolD^23^, ^24^. Moreover, two types of structures, a synthesis mode and an editing (proofreading) mode, were obtained^24^. This PolD structure provides a basis for understanding replisome structure and function. Then, as the essential core of the replisome, a helicase that unwinds the helix of the parent strand that serves as a template, a primase that synthesizes a primer on the unwound single‐stranded portion, and a replicase that extends the primer and synthesizes a nascent strand are combined together to form a functional complex structure. To elucidate how these constituent factors are arranged and how they efficiently cooperate with each other to promote the DNA replication reaction, we analyzed the interaction between each highly purified protein and identified each interaction site. Two molecules of PolD are linked to the CMG‐like helicase complex (Gan‐6Mcm‐2Gins51‐2Gins23) via Gins51, one of the GINS components. This structure suggested that PolD might be involved in both leading and lagging strand synthesis^25^. This interaction was found to be due to the binding of the N‐terminal domain of the DP1 subunit of PolD to the C‐terminal domain of the Gins51 subunit of GINS. To investigate the functional meaning of this interaction, we made a reconstitution system for leading strand synthesis and examined the effect of the interaction of the PolD–CMG‐like complex on nascent DNA synthesis. The helicase activity of the CMG‐like helicase was stimulated by increasing its single‐stranded DNA‐dependent ATPase activity in the presence of PolD^25^. These results for the first time identified and clarified the functional significance of a helicase‐polymerase binding mode, which is required in Archaea and probably in Eukarya, to synchronize parental DNA unwinding and nascent strand synthesis. This paper was elected as a “Breakthrough Article” in *Nucleic Acids Research* in 2022.

One more exciting result is that while PolD is stably bound to primase in the absence of DNA, PCNA is substituted with primase to bind PolD in the presence of dsDNA. Based on this binding partner exchange for PolD^26^, ^27^, a possible model is proposed, wherein after primase synthesizes a short primer, it subsequently synthesizes short DNA, after which primase passes the baton to PolD–PCNA, which continuously synthesizes nascent DNA strands^26^.

To deepen our understanding of the functional replication machinery, it is imperative to investigate whether the elongation mode of the replication machinery binds to other factors, for example, when a helicase unwinds a helix of the parent strands, topoisomerases are required to dissolve the distortions accumulated ahead of replication fork progression. The topoisomerase required for archaeal replication is thought to be TopoVI, and we are investigating the possibility that the dissociation of the double strand and the synthesis of the nascent strand have a connection with the elimination of distortion.

Regarding the molecular evolution of PolD, Ludvic Sauguet et al.^27^ found by crystal analysis of their truncated protein that DP2 has a 3D structure similar to that of RNA polymerase, which is a universally conserved transcriptase in living organisms. Based on this knowledge, we have inferred the ancestors of DNA polymerase with Eugene Koonin (National Institute of Health)^28^. Our scenario considers that the first transcription enzyme (RNAP) and the first replication enzyme (DNAP) evolved from a common ancestral protein that probably acted as RNA‐dependent RNA polymerase (RdRP). Therefore, LUCA replicase is a direct ancestor of the extant archaeal replicase PolD. Following the molecular evolutionary pathway in detail, two primitive RNA‐binding domains, double‐psi beta‐barrel (DPBB) and RNA recognition motif (RRM), have evolved into RdRP, RNAP, and DNAP. The DPBB pathway is associated with cellular evolution, and the RRM pathway is thought to be associated with the evolution of viruses and mobile genetic elements. This new evolutionary proposal for replication and transcription includes multiple opportunities for switching between RNA and DNA templates and products during evolution.

## ARCHAEAL RESEARCH IS A GOLDMINE FOR MOLECULAR BIOLOGISTS

I have had the fortune of many exciting discoveries of new proteins, which are different from homologs of the proteins related to DNA replication and recombinational repair from Bacteria and Archaea since I found PolD at the beginning of my research. I have thoroughly enjoyed my research journey, and I strongly feel that research on Archaea is a goldmine for molecular biology, to quote Patrick Forterre's words.

I heard the sad news that Prof. Woese passed away on December 30, 2012. People at the University of Illinois held a memorial symposium for Prof. Woese and the opening of the Carl Woese Institute for Genomic Biology in September, 2015 at the University Illinois campus. I attended it again and spent some time with people in the Archaea field. It was nice to find a monument of the phylogenetic tree showing three domains of life in the Institute (Figure 2C). Finally, I would like to thank Prof. Woese again for the special encouragement. He gave me the confidence to continue my research on these lines. In Japan, professors have to retire at 65 years of age, but I will continue to encourage and support young scientists studying the molecular biology of archaea by detailing the events from the dawn to the development period of archaeal molecular biology research. Just as stalwarts like Prof. Woese played a pivotal role in my research career, I would like to keep inspiring young researchers to follow their dreams and perform groundbreaking research that will clarify unresolved aspects of life and evolution.
